# A Microfluidic Chip for Cell Patterning Utilizing Paired Microwells and Protein Patterns †

**DOI:** 10.3390/mi8010001

**Published:** 2016-12-23

**Authors:** Chunlong Tu, Bobo Huang, Jian Zhou, Yitao Liang, Jian Tian, Lin Ji, Xiao Liang, Xuesong Ye

**Affiliations:** 1 Biosensor National Special Laboratory, Key Laboratory of BME of the Ministry of Education, Zhejiang University, Hangzhou 310027, China; tcl@zju.edu.cn (C.T.); hbb2013@zju.edu.cn (B.H.); zhouja@zju.edu.cn (J.Z.); liang1tao@zju.edu.cn (Y.L.); tianjian@zju.edu.cn (J.T.); 2 College of Biomedical Engineering & Instrument Science, Zhejiang University, Hangzhou 310027, China; 3 Department of General Surgery, Sir Run Run Shaw Hospital, College of Medicine, Zhejiang University, Hangzhou 310016, China; jilin@zju.edu.cn (L.J.); liangx@srrsh.com (X.L.); 4 State Key Laboratory of CAD&CG, Zhejiang University, Hangzhou 310027, China

**Keywords:** microfluidic, microfabrication, lab-on-a-chip, cell patterning, micro contact printing, cell capture, microwell, cell biology

## Abstract

Cell patterning has been widely used in research on fundamental cell biology and in applications such as tissue engineering, neuron network formation, cell based biosensor and drug screening. Although various methods have been developed, cell patterning in an enclosed microfluidic device at single cell level remains challenging. This paper describes a microfluidic device with microwells and protein patterns paired together in a single microchannel for an easy cell patterning. Cells captured in the microwells were positioned directly onto the protein patterns within 5 min and the patterning performance was successfully demonstrated using HeLa cells and human gallbladder carcinoma cells (SGC-996). Cells survived for 6 days in the microchannel. Cell attachment, migration, proliferation and cell colony formation were observed. Our device is free of topographic constraint for the patterned cells and no complex chemical modification to the substrate is needed, offering a simple, fast, and easy-to-operate way of patterning cells at single cell level in an enclosed microfluidic channel.

## 1. Introduction

The cell patterning technique is very useful to reveal fundamental cell physiological processes, such as cell migration [1,2], polarization [3,4,5], differentiation [6], proliferation [6,7] and cell signaling [5,6]. It is also widely applied in the research of tissue engineering [8,9], neuron network formation [10,11], cell based biosensor [12,13] and drug screening [14]. Research such as stem cell differentiation, cell heterogeneity and neuron science [15] shows great demands for cell patterning at single cell level [16].

Various approaches have been developed for patterning cells on a culture substrate, which can be classified into three types: physical patterning, chemical patterning and approaches combining both physical and chemical patterning. Certain types of physical cell patterning approaches such as inkjet cell printing [13,17], optical tweezers [18,19], dielectrophoresis [8,20,21] and laser-guided direct writing [22,23], position cells into specific locations directly, utilizing actively applied external forces. Although these methods are precise, the complicated experimental setup, potential damages to the cells due to the external forces and relatively low throughput limited their application. Other types of physical patterning approaches obtain cell patterns by capturing and confining cells in microfabricated mechanical structures such as microwells [6,14,24,25,26,27] and micro traps [28,29,30]. With optimized size and shape, these mechanical structures could perform high efficiency for cell patterning at single cell level [27,30]. However, there are still some limitations in the direct use of these mechanical methods in research such as cell migration, spreading, proliferation and polarization, as the topographic constraints that the mechanical structures bring may affect the growth of the cells. 

On the other hand, chemical cell patterning methods utilize selective attachment of randomly seeded cells on cell adhesive materials such as Poly-l-lysine (PLL) and adhesive proteins [10,31,32,33,34,35]. With the assistance of cell repellent materials to block the adjacent areas of the adhesive patterns, cells can be chemically confined in specific areas and form well defined patterns. Bashir’s group successfully demonstrated chemical cell patterning on fully suspended resonant sensors for measurement of cell mass during their growth [33], showing great versatility of chemical cell patterning. Although chemical cell patterning is free of topographic constraints, it usually needs complex chemical modifications, such as pre-coating and back filling of cell repellent materials. These chemical modifications may cause a residual toxicity, and are difficult for biologists. Additionally, chemical constraint applied by cell repellent materials prevents the revealing of the cells’ natural characteristics, especially in cell migration and proliferation applications. Some other chemical approaches pattern cells without cell repellent materials [15,36,37]. Millet et al. fabricated patterns and gradients of adhesive proteins by microfluidics-based substrate deposition, which successfully guided neuronal development [37]. These approaches were usually used in neuron science research, as neurons are known to be fragile and hard to attach to the substrate without adhesive materials.

Besides, cell patterning methods combining physical and chemical approaches have also been developed [38,39,40,41]. Ostuni et al. reported a convenient method for cell patterning using microwells coated by fibronectin, a commonly used cell adhesive protein [38]. Cells deposited, attached and grew on the adhesive area in the microwells, while the microwells limited their spreading, migration and proliferation. Rodriguez’s group recently reported a novel single cell patterning system using hydrodynamic traps and protein patterns in a microfluidic device [40]. However, the fabrication of the delicate sieve-like cell traps is complex. The micro trap will restrict the growth of the cells if they are not removed after cell attachment, while the removing step may bring damages and risks of contamination to the cells.

**Figure 1:**
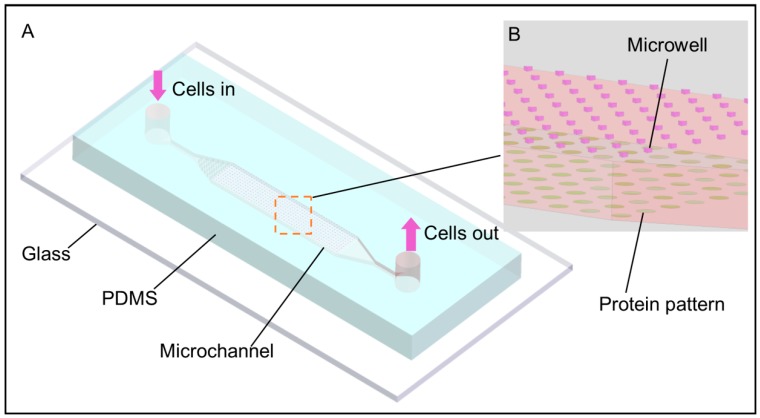
Schematic of the microfluidic chip with protein patterns paired with triangular microwells. Not to scale. Cells are captured within the microwells in the first step while these wells are flipped onto the floor. After flipping again, captured cells leave from the microwells and fall onto the corresponding protein patterns under gravity. Both the inlet and outlet are sealed before the second flipping to minimize possible perturbations of flow inside the channel. (**A**) Schematic of the whole chip; (**B**) a tilted, magnified view of the channel structure. The protein patterns (green colored) on the floor are precisely paired with the microwells (red colored) on the ceiling.

Herein, we developed a simple microfluidic chip for cell patterning, combining both physical microwells and chemical protein patterns in the same enclosed microfluidic channel. Microwells on the ceiling were designed for rapid and efficient cell capture at single cell level (or small numbers of cells), and protein patterns on the floor were for preferential cell attachment and growth (Figure 1). Cells were first loaded into the channel and captured by the microwells with the chip facing down; captured cells were then released from microwells and settled onto the protein patterns under gravity after a simple flipping of the chip. The whole cell patterning operation can be finished in 5 min. Two cancer cell lines—HeLa and human gallbladder carcinoma cells (SGC-996)—were used to demonstrate and analyze the patterning performance of our chip. Cell migration, cell proliferation and colony formation of both types of cells were successfully observed. With a main strategy of “capturing and releasing”, cells were positioned and patterned without complicated experiment setup or external forces except gravity, compared with inkjet-based, optical and dielectrophoresis approaches. Our device is free of topographic constraint compared with physical patterning approaches utilizing mechanical structures, and has no chemical confinement in contrast to some chemical patterning approaches. Furthermore, both microwells and micro contact printing (μCP) used in our device can be simply implemented in most biology laboratories after the fabrication of the master and no chemical surface modifications or specific experiences are needed, making our device a simple, fast and easy-to-operate method of cell patterning in a microfluidic device.

## 2. Materials and Methods

### 2.1. Micro Contact Printing of Protein on the Substrate

**Figure 2:**
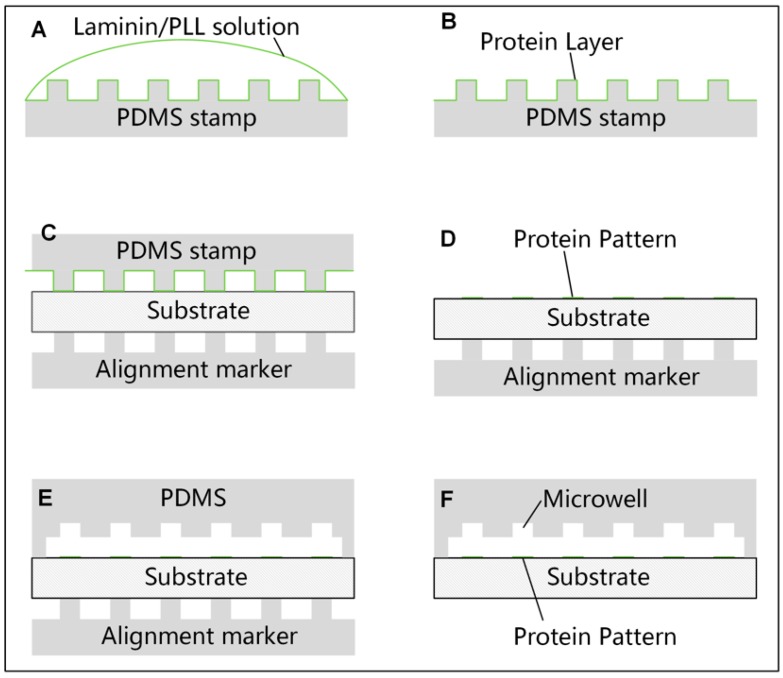
Fabrication of the of the microfluidic device with paired microwells and protein patterns. Schematics were not drawn to scale. (**A**,**B**) Stamp incubation with the protein solution; (**C**,**D**) Printing protein patterns on the substrate; (**E**,**F**) Protein patterns and microwells pairing with the help of a polydimethylsiloxane (PDMS) alignment marker.

Micro contact printing (μCP) was employed to print Poly-l-lysine (PLL) or protein on the substrate of the microchannel. Procedures were modified according to published method [32]. A polydimethylsiloxane (PDMS) (DC-184, Dow corning, Midland, MI, USA) slab with an array of pillars fabricated by standard lithography was used as a stamp. The pillars on the stamp were 120 µm in diameter and 40 µm in height. A glass coverslip (80340-3610, Citotest Labware Manufacturing Co., Ltd., Haimen, China) with a thickness of 150 µm was chosen as the substrate. Coverslips were sonicated in ethanol and deionized (DI) water sequentially and N_2_ dried before use. The surface of the PDMS stamp was first treated with O_2_ plasma for 30 s to facilitate the spread and infiltration of the protein solution. Then, a drop of PLL (P4707, Sigma-Aldrich, St. Louis, MO, USA) solution at the concentration of 50 µg/mL, or Laminin (L2020, Sigma-Aldrich) solution at the same concentration, was added onto the stamp and incubated for 30 min at room temperature (Figure 2A). The stamp was periodically observed to not get dried during the incubation. After removing the remaining solution, a mild wash with 0.01 M phosphate-buffered saline (PBS) was applied to the coated stamp for two times. The stamp was then swept by N_2_ mildly and left to dry in a clean hood for 5 min (Figure 2B). To mark the position of the protein patterns printed on the substrate precisely, another PDMS slab with the same pillar array as the stamp was reversible bonded on the other side of the coverslip as a marker during the fabrication process (Figure 2C–E). After treating the coverslip with O_2_ plasma for 1 min, the stamp was aligned with the marker under a microscope and fully contacted to the substrate for 5 min before removing. An inverted microscope customized as a simple triaxial alignment system was employed to facilitate the alignment.

Fluorescein isothiocyanate labeled poly-l-lysine (PLL-FITC) (P3069, Sigma-Aldrich) was used to optimize the micro contact printing procedures. Fluorescence images of different samples prepared with different procedures were acquired by the same microscope and electron multiplying charge coupled device (EMCCD) camera with the same settings. The florescence intensity and uniformity were analyzed to evaluate the qualities of the patterns.

### 2.2. Fabrication and Preparation of the Microfluidic Chip

Standard soft lithography was employed to fabricate the microfluidic channel with microwells. First, a layer of SU-8 photoresist (3050, MicroChem Corp., Westborough, MA, USA) with a height of 50 µm was patterned on a polished Si-wafer to form the mold of the microchannel. Then, a second layer of SU-8 photoresist with a height of 40 µm was patterned on the first layer to form the microwells. The microwells were designed to be equilateral triangular with three different sizes (40, 50, 60 µm in side length, respectively) in a 20 mm × 2 mm rectangular microchannel. Uncured PDMS at a weight ratio of 10:1 (base and curing agent) was cast on the mold and cured at 80 °C on a hotplate for 1 h. Cured PDMS slabs were cut by a scalpel and carefully peeled off from the mold manually. After that, a flat head needle was used to punch through the PDMS slab to form the inlet and outlet of the microchannel. Finally, PDMS debris and dust on the PDMS slab was washed away by ethanol and DI water in sequence. The PDMS slab was then aligned with the marker with the help of a customized microscope and bonded to the substrate with former printed protein patterns after an O_2_ plasma treatment for 30 s at 29.6 W power (PDC-002, Harrick Plasma Inc., Ithaca, NY, USA) (Figure 2D,E). To prevent the protein patterns on the substrate from being damaged, a piece of PDMS was covered on the protein patterns before the plasma treatment.

The microchannel was incubated by 5 wt % bovine serum albumin (BSA) (V900933, Sigma-Aldrich) solution for 30 min at room temperature to prevent cells from attaching to the microwells immediately [42]. Bubbles in the microchannel, especially in the microwells, were carefully removed by applying an occasionally changing flow of BSA solution. The microchannel was then washed with 0.01 M PBS and stored in a clean hood before use.

### 2.3. Cell Culture and Cell Suspension Preparation

HeLa cells were cultured in complete Dulbecco’s Modified Eagle Medium (DMEM) (Cat. No. 11995-065, Gibco™, Thermo Fisher Scientific, Waltham, MA, USA) supplemented with 10 vol % fetal bovine serum (FBS) (Cat. No. 10099-133, Gibco™, Thermo Fisher Scientific) and 1 vol % Penicillin–Streptomycin (Cat. No. 15140-148, Gibco™, Thermo Fisher Scientific) in a 25 cm^2^ polystyrene flask (Cat. No. 430639, Corning Inc., Corning, NY, USA) at 37 °C and 5% CO_2_ in a humidified incubator (BB15, Thermo Fisher Scientific). SGC-996 cells were cultured in complete Roswell Park Memorial Institute (RPMI) 1640 medium (Cat. No. 11875-119, Gibco™, Thermo Fisher Scientific) supplemented with 10 vol % FBS and 1 vol % Penicillin–Streptomycin in the same culture condition.

All cells were passaged at 70%–80% confluence using 0.05% Trypsin-EDTA (Cat. No. 25300-054, Gibco™, Thermo Fisher Scientific) under the common passage protocol. Briefly, cells were first rinsed twice with pre-warmed PBS, then 1 mL pre-warmed Trypsin-EDTA solution was added. After an incubation in a 37 °C incubator for 5 min, 1 mL complete culture medium was added to stop the trypsinization. Cell suspension was then transferred into a conical tube and centrifuged for 5 min at 1000 rpm (STR16, Thermo Fisher Scientific). Supernatant was discarded and cells were resuspended in complete culture medium and reseeded. Cell concentration was determined using a hemacytometer and was adjusted to the required concentration. Hoechst 33258 (Cat. No. H3569, Molecular Probes, Eugene, OR, USA) was used to stain the nucleus of cells according to the operation manual provided by the manufacturer. Briefly, cells were incubated in Hoechst 33258 solution at the concentration of 2 µg/mL at 37 °C and 5% CO_2_ for 20–30 min before passage. Cells loaded into our chip were resuspended in D-Hanks buffer (GNM-14175, Genom, Hangzhou, China) with 0.02 wt % EDTA (E6758, Sigma-Aldrich).

### 2.4. Cell Loading and Experiment Setup

**Figure 3:**
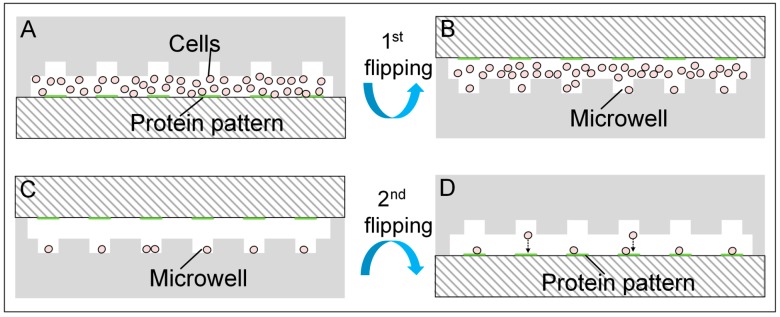
Cell loading processes. Schematics were not drawn to scale. (**A**) Cell suspension was loaded into the microchannel with the chip facing up; (**B**) cells settled in the microwells after the first flipping of the chip; (**C**) cells settled in the microwells after a fast flow of 20 μL/min; (**D**) cells dropped on the protein patterns after the second flipping of the chip.

A syringe driven by a syringe pump (NE-4002, New Era Pump Systems Inc., Farmingdale, NY, USA) was connected to the inlet of the chip via a 1/16” Peek Teflon tubing (Upchurch Scientific, Oak Harbor, WA, USA) and suitable fittings (Upchurch Scientific) to apply flows into the microchannel. Cell suspension at a concentration of 4 × 10^6^ cells/mL was gently pipetted with a fine pipette tip and aspirated into a 200 μL pipette tip soon after preparation. The tip was then inserted into the outlet of the chip and a negative flow at the speed of 20 μL/min from the inlet was applied to load cells into the microchannel (Figure 3A). The tip was removed after full filling of the microchannel and the chip was flipped for the first time with the upside facing down (Figure 3B). Subsequently, a positive flow from the inlet to the outlet was driven into the microchannel at the speed of 2 μL/min for 2 min to improve the capture efficiency [25]. Then, the speed was increased to 20 μL/min for 2 min to wash away the uncaptured cells (Figure 3C). After that, the tubing at the inlet was removed and both the inlet and the outlet were carefully sealed with PDMS films to stop unwanted flow. Finally, the chip was flipped again to release captured cells out of the microwells onto the protein patterns under gravity (Figure 3D).

### 2.5. Cell Culture in the Microfluidic Chip

After cell loading, the chip was packaged into a petri dish with Parafilm (Bemis, Neenah, WI, USA) and transferred into the incubator. To slow down the evaporation of the medium, 1 mL of sterile DI water was added into the petri dish. Cells in the microfluidic chip were incubated for 12 h before the first change of the medium. PDMS films at the inlet and outlet of the chip were carefully removed and 30 µL of pre-warmed complete medium was added to the inlet. The medium in the outlet was carefully removed with a pipette two times to completely change the medium in the microchannel. Both the inlet and the outlet were then covered by PDMS films again. The chip was packed in a petri dish and transferred back into the incubator. The medium was replaced with flash medium every 12 h in the following days. Cells were imaged every day to analyze the growth.

### 2.6. Imaging and Cell Analysis

An inverted epi-fluorescence microscope (DMI4000, Leica, Wetzlar, Germany) equipped with a high-speed EMCCD camera (iXon ultra 897, Andor Technology Ltd., Belfast, UK) was used to observe the cells and acquire all the images. ImageJ^®^ (1.48v, National Institutes of Health, Bethesda, MD, USA) was used for image processing and analysis. Line scans of the fluorescence intensity were used to evaluate the quality of the PLL-FITC patterns. Cell size was measured manually with the help of ImageJ^®^. To analyze the capture efficiency, cells were counted manually.

## 3. Results and Discussion

### 3.1. Cell Patterning Microfluidic Device with Paired Microwells and Protein Patterns

A microfluidic cell patterning device with precisely paired microwells and protein patterns in the same microchannel was fabricated for rapid single cell patterning as shown in Figure 1A. Microwells on the ceiling of the microchannel were used to capture cells while the protein patterns on the floor were used to support the adhesion and growth of cells. Equilateral triangular microwells were used for efficient single cell capture [27] and three designs of microwells with side lengths of 40, 50 and 60 μm and a depth of 40 μm were fabricated according to cell sizes used in this work. PLL and Laminin, which are commonly used to facilitate the adhesion of cells on substrate, were pre-patterned on the floor by µCP. The protein patterns were designed to be round with a diameter of 120 μm. The microwells and the protein patterns were paired correspondingly as shown in Figure 1B. The distance between the centers of two adjacent microwells or protein patterns was 200 μm, which was long enough to promote a sufficient separation of cells from each other and was also close enough for cell–cell interaction in the early days after cell loading [6]. Cells first captured in the microwells were positioned on the protein patterns undergoing gravity, simply by flipping the chip [25], and no extra releasing operation such as removing the capture structures [40], was needed. 

**Figure 4:**
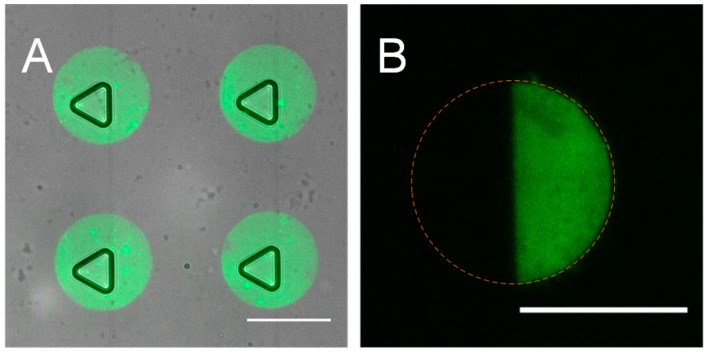
Device fabrication. (**A**) Overlapped images of paired fluorescein isothiocyanate labeled poly-l-lysine (PLL-FITC) patterns and microwells within a microchannel. Scale bar is 100 μm; (**B**) fluorescence image of a PLL-FITC pattern after a 30 s O_2_ plasma treatment with the right side covered by a piece of PDMS. Dotted circle shows the original shape of the pattern before O_2_ plasma treatment. The protected right side maintained high fluorescence while the exposed left side was totally damaged. Scale bar is 100 μm.

There are two main challenges in the fabrication of our device. First, it is not easy to achieve a precise alignment of the microwells and the protein patterns directly, due to the transparency of the patterns. We employed a PDMS slab as a marker to assist this process (Figure 2C–E). The PDMS marker had the same structure as the PDMS stamp to guarantee the accuracy and a proper size (50 mm × 20 mm) for easy handling. Figure 4A showed the top view of the completed device with precisely paired microwells and PLL-FITC patterns. Another challenge is to prevent the protein patterns being damaged during the oxygen plasma bonding, as early work has shown that the plasma could destroy the patterned protein [39]. The printed protein patterns were covered by another PDMS slice initially to avoid potential damage. According to our experiment, PLL-FITC patterns protected by the PDMS slab maintained a high fluorescence after a 30 s O_2_ plasma treatment, while exposed patterns disappeared, indicting a sufficient protection by this method (Figure 4B).

### 3.2. Optimization of the Micro Contact Printing

The parameters of μCP were modified from those in the literature [32]. Substrate material, substrate treatment and stamp preparation were tested and optimized. As the protein patterns printed by μCP only have a thickness of several nanometers [43], and are transparent under a normal microscope, direct evaluation of their quality is difficult. We printed PLL-FITC as a visible indicator by different μCP procedures and evaluated their qualities, taking the fluorescence intensity and uniformity as two criteria. Patterns with high and uniform fluorescence intensity were considered as high-quality. Indirectly, the optimized procedures were elected and we assumed that they were also applicable for PLL and Laminin.

**Figure 5:**
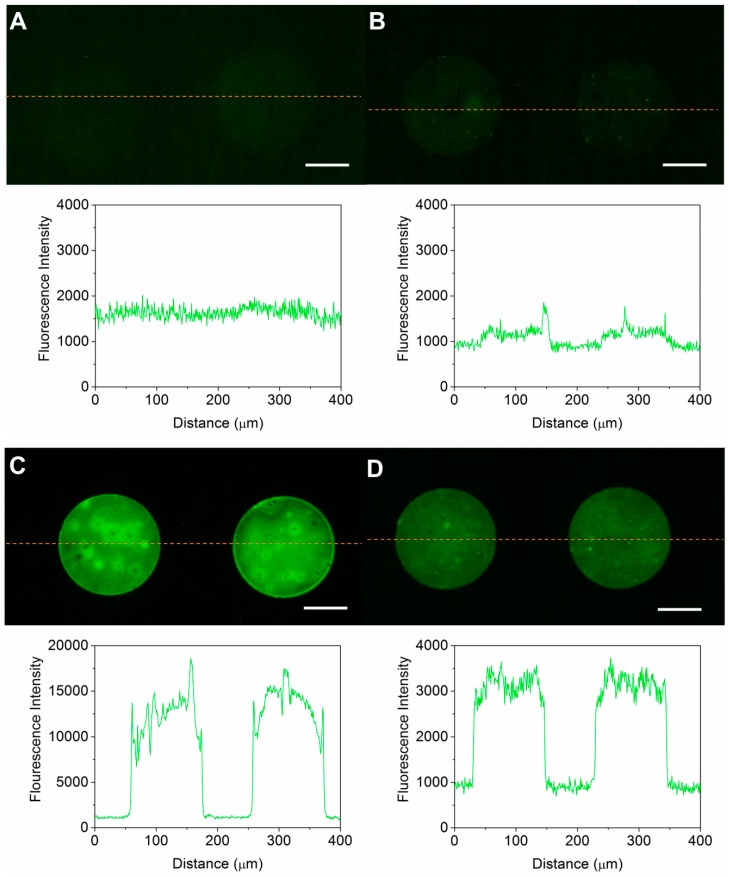
Fluorescence images and corresponding line scans showing the quality of the micro contact printing (μCP) process using PLL-FITC by different procedures. All the results were obtained with the same settings of the microscope and electron multiplying charge coupled device (EMCCD) camera. (**A**) Micropatterns on an O_2_ plasma-treated PDMS substrate; (**B**) micropatterns on a glass substrate without O_2_ plasma treatment; (**C**) poor uniformity of micropattern on an O_2_ plasma-treated glass substrate using a non-washed stamp, with an average fluorescence intensity of 12,922 ± 2278; (**D**) good uniformity and high contrast ratio of micropatterns on an O_2_ plasma-treated glass substrate using a mildly washed stamp, with an average fluorescence intensity of 3114 ± 262. Scale bars are 50 μm.

Glass was preferred over PDMS as a substrate in terms of patterning quality (Figure 5A,C). The fluorescence intensity from glass was much higher. The efficiency of the material transfer is determined by hydrophobicities and protein bonding capabilities of the stamp and the substrate [44,45]. A high protein bonding capability of the substrate and lower capability of the stamp is favorable for the transfer efficiency. Also, a sufficient O_2_ plasma treatment was necessary to enhance the transfer of PLL-FITC onto the glass substrate (Figure 5B,C). O_2_ plasma treatment makes the glass substrate more hydrophilic and facilitates the bonding of PLL-FITC with the glass. Besides, direct use of the stamp without washing after incubation reduced the uniformity of the protein patterns (Figure 5C). This was because residual PLL-FITC solution on the stamp may concentrate into considerable high concentration and even crystallize in some areas when the stamp was left to dry. A mild wash to the stamp by PBS after coating showed a significant improvement of pattern uniformity (Figure 5D), despite the intensity declining significantly, because PBS dissolved the residual PLL-FITC of high concentration, leaving a more uniformly coated surface. As a result, we adopted O_2_ plasma-treated glass substrates and mild washed stamps for the subsequent experiments.

**Figure 6:**
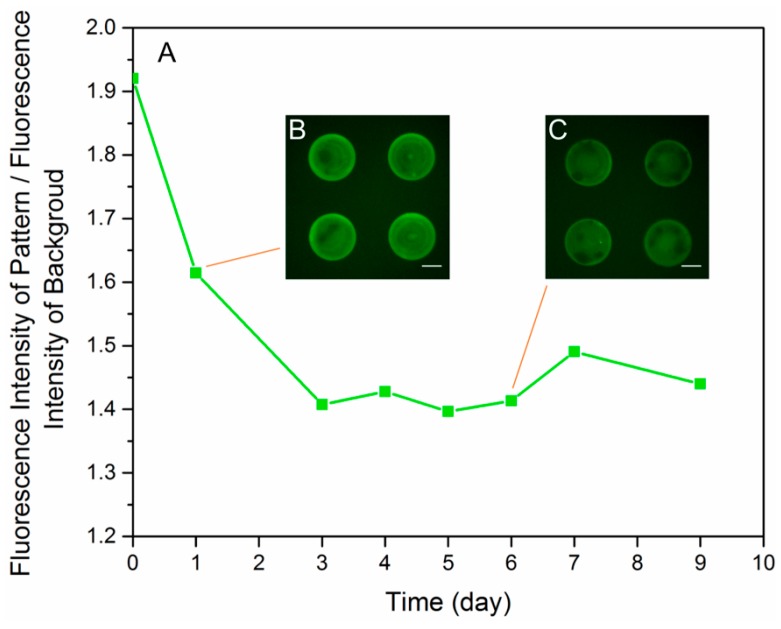
Lifespan of the PLL-FITC patterns in the complete culture medium. (**A**) Curve of the ratio of the fluorescence intensity of the PLL-FITC patterns to the fluorescence intensity of the background over 9 days; (**B**) fluorescence image of PLL-FITC patterns on Day 1; (**C**) fluorescence image of PLL-FITC patterns on Day 6. Images were taken with the same settings of the microscope and EMCCD camera. Scale bars are 50 μm.

The lifespan of the protein patterns was tested to be at least 7 days. PLL-FITC patterns printed by the optimized procedures were immersed in complete culture medium in the incubator for a week. Fluorescence images of the sample were taken every 24 h with the same setting of the microscope and EMCCD camera. The contrast, defined as the ratio of the intensity of the PLL-FITC patterns and the intensity of the background, was taken as a criterion (Figure 6A). Degeneration of the fluorescence intensity was observed in the first three days, as the contrast dropped from 1.9 to 1.4 (Figure 6B). This degradation was possibly due to the quenching of the fluorescence group and diffusion of PLL-FITC into the medium. Despite the degeneration, the contrast still remained at around 1.4 times in the following days and clear patterns could be observed (Figure 6C). The result showed that PLL-FITC patterns on the substrate in the culture mediums degenerated over time while maintaining an adequate level after 7 days.

### 3.3. Cell Capture Performance Demonstrated with HeLa Cells and SGC-996 Cells

**Figure 7:**
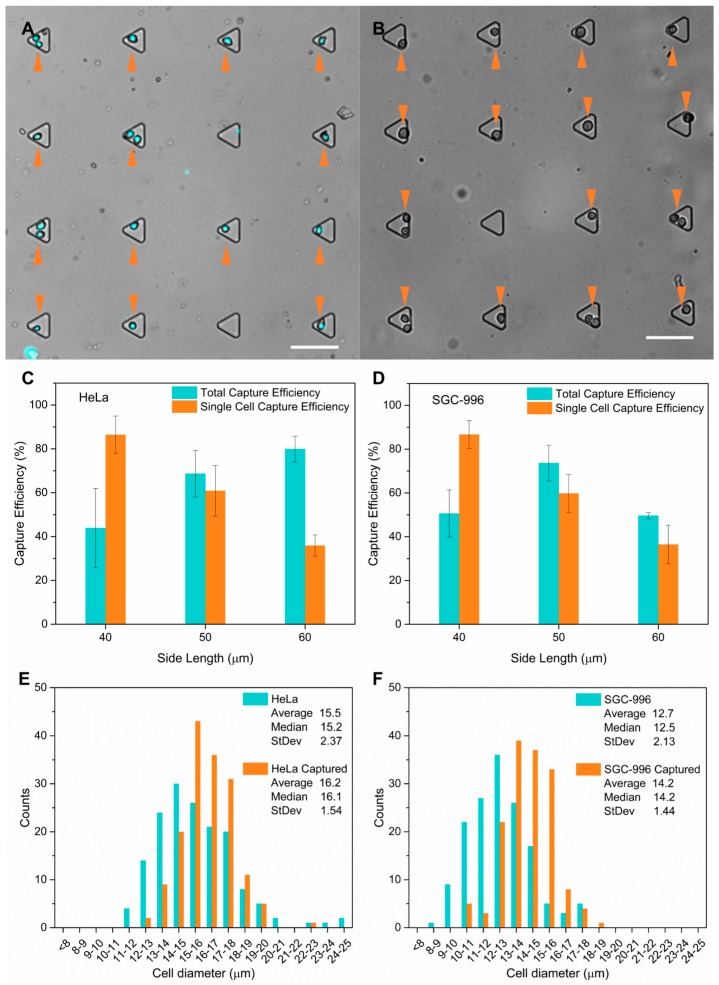
Cell capture performance. HeLa and human gallbladder carcinoma cells (SGC-996) suspension at a concentration of 4 × 10^6^ cells/mL was used, respectively. (**A**) HeLa cells stained with Hoechst 33258 (cyan colored) were captured in the microwells after washing. Scale bar is 100 μm; (**B**) HeLa cells were released from the microwells and fell on the protein patterns (non-fluorescently labeled). Note that there was a distance of 50 μm from the microwells to the paired patterns, and the focus plane was on the microwells; the blurred cells indicated that they had fallen out of the microwells. Scale bar is 100 μm; (**C**,**D**) cell capture efficiencies of triangle microwells with three side lengths. The total capture efficiency for HeLa cells increased with the side length while a peak efficiency was achieved with 50 μm side length microwells for SGC-996 cells. The single capture efficiency for both types of cells dropped dramatically when the side length increased from 40 to 60 μm; (**E**,**F**) size distributions of the original cells and captured single cells by 50 μm side length microwells of HeLa and SGC-996 cells. Diameters of captured HeLa single cells concentrated in a range of 15–18 μm and 13–16 μm for captured SGC-996 cells, while the origin cells distributed in wider ranges. The average diameter of the captured single cells of both types increased while the standard deviations dropped, implying a promoted uniformity of cell size.

Cells were patterned by four steps with our device (Figure 3): (1) cell suspension loading; (2) first flipping of the chip to capture cells; (3) a fast flow to wash away uncaptured cells; and (4) second flipping to release the cells to the patterns. Figure 7A shows the Hoechst 33258 stained HeLa cells captured in the microwells after step (3), and Figure 7B shows HeLa cells on the protein patterns after step (4). The whole cell patterning process can be finished in 5 min—faster than common passive cell patterning methods—by selective attachment of cells, and by methods including inkjet printing, optical tweezers, dielectrophoresis and laser-directed cell writing. Although the microwells were incubated with BSA prior to use, cells might be retained in the microwells if step (2) and (3) take a long time. According to our experiment results, more than 95% of the captured cells could be released if the operation time of step (2) and (3) was less than 3 min.

HeLa and SGC-996 cells were used to characterize the capture performances of our devices with three types of microwells with side lengths of 40 µm, 50 µm and 60 µm, respectively. Our results showed that capture efficiency was influenced by both sizes of the microwells and the cells. Images of three random areas of the microwell arrays were taken and capture efficiency was analyzed after cell loading. For each size of microwell and each type of cell, five batches of experiments were conducted, and in total about 500 microwells were investigated for each batch of experiment. We calculated the total capture efficiency (η_tot_), which was defined as the ratio of microwells occupied by one or more cells over total wells, and the single cell capture efficiency (η_s_), which was defined as the ratio of microwells occupied by a single cell among all the microwells occupied by one or more cells. For HeLa cells, when the side length of the microwells increased from 40 to 60 µm, the average total capture efficiency went up from 43.9% ± 17.9% to 79.8% ± 5.8%, while the single cell capture efficiency dropped dramatically from 86.4% ± 8.5% to 35.9% ± 4.9% (Figure 7C). For SGC-996 cells, while η_s_ showed a similar decrease trend as that of HeLa cells, η_tot_ first increased followed by a decrease when the side length of the microwells increased from 40 to 60 µm (Figure 7D). According to our measurements, the average diameter of HeLa cells (15.5 ± 2.37 µm) was about 3 µm larger than that of SGC-996 cells (12.7 ± 2.13 µm). Consequently, the highest η_tot_ with HeLa cells (79.8% ± 5.8%) was achieved in the largest microwells with a side length of 60 µm and the microwells with the highest η_tot_ for SGC-996 cells (73.7% ± 8.1%) were 50 µm in side length. Based on this, we presume that larger cells need microwells with longer side length for the highest η_tot_, and the varied capture efficiency between the two types of cells is a result of their different sizes. In terms of single capture efficiency, trends of the two types of cells were consistent, indicating that the size of the microwells rather than the size of cells played a dominant role here. These results showed that the capture efficiency of our device was dependent on both the sizes of the microwells and the cells. As a compromise between total and single capture efficiency, we adopted microwells with 50 µm side length in the subsequent cell patterning experiments for both types of cells.

We further investigated the influence of the capture process to the size distribution of the cells. We analyzed the diameter distribution of single cells captured by microwells with 50 µm side length and compared it with the original distribution (Figure 7E,F). A total of 158 captured HeLa cells, 152 captured SGC-996 cells and the same number of original cells were measured, respectively. Diameters of captured HeLa cells concentrated in a range of 15–18 μm, and 13–16 μm for SGC-996 cells, while the original diameter of both types of cells distributed in wider ranges. Besides, for both types of cells, the average diameter of captured cells increased, while the standard deviation dropped, from 15.5 ± 2.37 µm to 16.2 ± 1.54 µm for HeLa cells (Figure 7E), and 12.7 ± 2.13 µm to 14.2 ± 1.44 µm for SGC-996 cells (Figure 7F), indicating an improved uniformity of the size. Based on these observations, we presume that cell capture in our device is not a random process. During the cell loading process, a fast flow was applied to wash away the uncaptured cells. This flow also applied nonnegligible influences to cells captured in the microwells. For microwells with specific side length, cells in the microwells with improper size would be brought out by the fast flow more easily, contributing to the concentrated size distribution, increased average diameter and improved uniformity of the captured cells. The shifts of these cell size distributions imply that microwells with specific side length are prone to capture cells with relevant size, which may give our device the potential ability of cell size dependent screening.

To quantify the final patterning yield and reproducibility of the device with 50 µm side length microwells, we calculated the ratio of the number of patterns occupied by cells to that of the total patterns (η_p_). Only cells that fell in the circular patterns with a diameter of 120 µm were considered to be well patterned. For each type of cells, three batches of experiments were conducted and three areas were picked randomly for the analysis. For HeLa cells, the average η_p_ of three experiments was 65.7% ± 10.1% (52.1%, 68.8% and 76.3%), and 71.1% ± 11.5% (56.3%, 72.9% and 84.2%) for SGC-996 cells. These results were consistent with the total capture efficiency of the same device for both types of cells (68.7% ± 10.6% for HeLa cells and 73.7% ± 8.1% for SGC-996 cells). Except the capture efficiency, two issues may decrease the patterning yield. The first one was that a few cells might be retained in the microwells, which could be overcome by a fine pretreatment of the microwells using BSA solution and a short operation time when capturing cells. Another one is that cells might be moved out of the protein patterns when falling from the microwells by the unwanted flow if the inlet and the outlet were not fully sealed. The circular patterns were designed larger than the microwells to counteract the slight position shift of the cells during the alignment, which was measured to be less than 30 µm in our device (Figure 7B). According to our observation, microwells 50 µm in side length with paired protein patterns 120 µm in diameter worked well.

### 3.4. Cell Patterning Performance with HeLa and SGC-996 Cells

**Figure 8:**
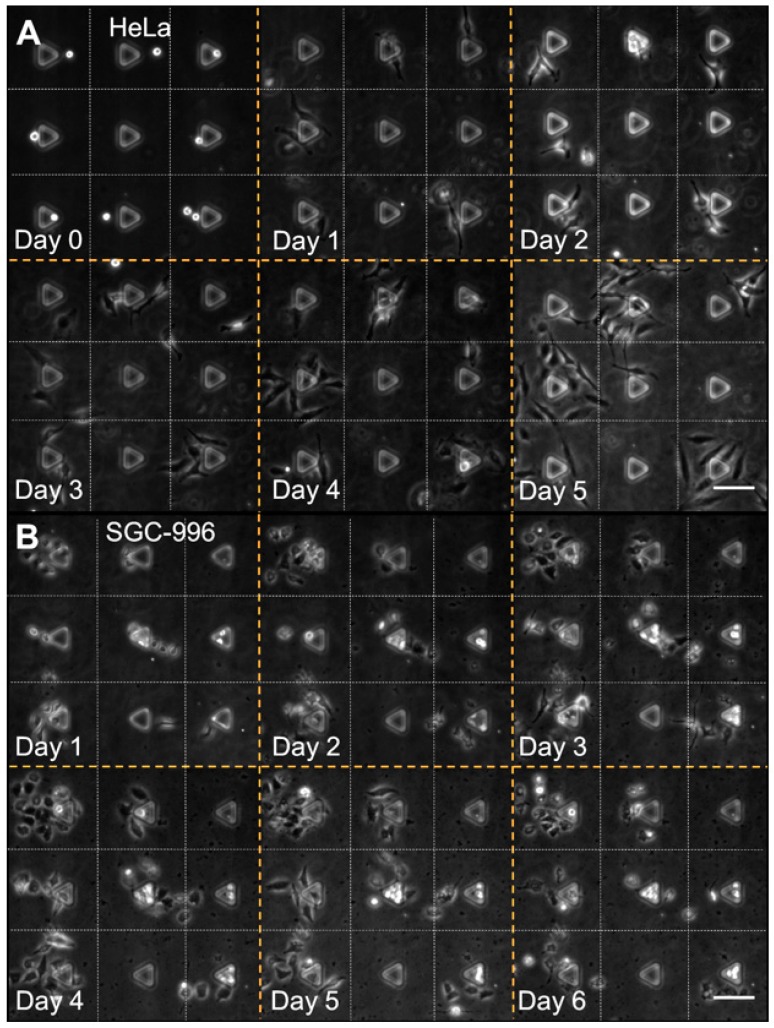
Time-lapse phase contrast images of HeLa cells and SGC-996 cells growing on PLL patterns in our device. (**A**) Patterning performance with HeLa cells on the PLL patterns (non-fluorescently labeled) from DAY 0 to DAY 5. Scale bar is 100 μm; (**B**) patterning performance with SGC-996 cells on the PLL patterns (non-fluorescently labeled) from DAY 1 to DAY 6. Scale bar is 100 μm.

To demonstrate the cell patterning performance of our device, HeLa and SGC-996 cells were successfully patterned at single cell level in an array of round patterns with a diameter of 120 µm and spacing of 200 µm. Time-lapse images showed that HeLa and SGC-996 cells successfully survived for at least 6 days in our device and the migration, proliferation and colony formation of both cell types were observed (Figure 8). After loading, cells were cultured in the microchannel in the incubator with FBS-free medium for 12 h before the medium in the microchannel was replaced with fresh complete culture medium. During the first 12 h, most of the cells completed the attachment and some of them began to spread and proliferate on the protein patterns. As there was no FBS in the medium, it was mainly the protein patterns that supported the attachment of the cells as extracellular matrix (ECM) materials. Then, the medium was replaced every 12 h with complete culture medium supplemented with 10 vol % FBS to support cell growth. The growth could be loosely divided into two typical stages for both cell types. In the first stage (Figure 8, Day 0 to Day 2), cells attached to the protein patterns and divided into small cell clusters containing two or more cells; a few cells migrated out of the patterns. In the second stage (Figure 8, Day 3 to Day 5), the proliferation sped up obviously and cell colonies formed gradually. Besides, cells also began to migrate out of the protein patterns to establish connections with adjacent cells in this stage. According to these results, cell–material interactions might be essential to cell attachment and proliferation during the first stage, and cell–cell interactions of adjacent cells could play a more important role in the second stage.

Compared with cell patterning devices based on physical structures, such as microwells and micro sieves, our device is free of topographic constraint in the micro environment around patterned cells, which may hinder the spreading, migration and proliferation. Also, the absence of topographic constraint brings a higher medium exchange efficiency, which plays an important role in cell–cell interaction by diffusible signaling [6,46]. Furthermore, cells were first captured and then transferred onto the protein patterns in our device, which is different from devices utilizing selective attachment of cells. No cell attachment repellent materials such as poly(ethylene glycol) (PEG) were needed to chemically restrict cells in specific areas, so that complex chemical modifications were avoided. Cells can grow more freely and more closely, reflecting characteristics in natural conditions. According to our observation, HeLa presented longer cell bodies, and more long pseudopodia protruded out (Figure 8A, Day 1), while SGC-996 maintained polygonal cell bodies with a few short pseudopodia (Figure 8B, Day 1). Besides, HeLa cells showed a more aggressive migration capability than SGC-996 cells and were more prone to connect with each other (Figure 8A, Day 2), while SGC-996 cells tended to form cell colonies by themselves (Figure 8B, Day 2), suggesting that HeLa cells may have stronger cell interactions with each other. In addition, different characters were also observed among individual cells of the same cell type. These results demonstrated the ability of our device in the study of cell attachment, migration, proliferation, colony formation and cellular heterogeneity.

The main strategy of our device can be summarized into “capturing–releasing”, which takes both advantages of microwells and protein patterns. Microwells provide an easy way to position cells into a desired layout at single cell level with a high throughput, and protein patterns provide suitable extracellular matrix materials for cells to undergo biological processes. An easy way that one may think of in the first place is to pattern cells in a substrate coated with uniform cell adhesive materials. Compared with our device, this method lacks intrinsic capacities of the protein patterns in cell biological research such as cell–material interaction, cell shape engineering and neuron network formation, resulting in a limited application. In this work, we used spaced round patterns of PLL and Laminin as a demonstration. The layout and shape of the patterns can be easily verified by utilizing different stamps. Furthermore, different materials, including cell repellent materials, can be patterned to enhance the ability of our device in the research of cell–material interaction and guided neuron network formation in our future work.

## 4. Conclusions

In conclusion, we successfully demonstrated a simple, fast and precise way for cell patterning in a microfluidic chip without utilizing cell selective attachment or cell repellent materials. The microchip incorporated with a paired array of microwells and protein patterns was fabricated following the optimized procedures to capture and transfer cells into designed positions. HeLa and SGC-996 cells were patterned on the PLL or Laminin patterns in 5 min at single cell level and survived for 6 days. Cell attachment, migration, proliferation and colony formation for both types of cells were observed. We also analyzed the influence of the sizes of microwells and cells to the capture performance, which is helpful for research using other cell types. Without topographic constraint to the patterned cells and complex chemical modifications, this simple, fast and efficient cell patterning method provides a convenient approach for cell biology research which are sensitive to the initial cell position and extracellular environment, such as single cell analysis, cell–material interaction, cell–cell interaction, cell co-culture, drug screening, cell colony formation and guided formation of the neuron network.
